# One Clinician Is All You Need–Cardiac Magnetic Resonance Imaging Measurement Extraction: Deep Learning Algorithm Development

**DOI:** 10.2196/38178

**Published:** 2022-09-16

**Authors:** Pulkit Singh, Julian Haimovich, Christopher Reeder, Shaan Khurshid, Emily S Lau, Jonathan W Cunningham, Anthony Philippakis, Christopher D Anderson, Jennifer E Ho, Steven A Lubitz, Puneet Batra

**Affiliations:** 1 Data Sciences Platform The Broad Institute of Harvard and MIT Cambridge, MA United States; 2 Department of Medicine Massachusetts General Hospital Harvard Medical School Boston, MA United States; 3 Cardiovascular Research Center Massachusetts General Hospital Boston, MA United States; 4 Cardiovascular Disease Initiative The Broad Institute of Harvard and MIT Cambridge, MA United States; 5 Demoulas Center for Cardiac Arrhythmias Massachusetts General Hospital Boston, MA United States; 6 Division of Cardiology Brigham and Women’s Hospital Boston, MA United States; 7 Eric and Wendy Schmidt Center The Broad Institute of Harvard and MIT Cambridge, MA United States; 8 Department of Neurology Brigham and Women’s Hospital Boston, MA United States; 9 Henry and Allison McCance Center for Brain Health Massachusetts General Hospital Boston, MA United States; 10 Center for Genomic Medicine Massachusetts General Hospital Boston, MA United States; 11 CardioVascular Institute and Division of Cardiology Department of Medicine Beth Israel Deaconess Medical Center Boston, MA United States

**Keywords:** natural language processing, transformers, machine learning, cardiac MRI, clinical outcomes, deep learning

## Abstract

**Background:**

Cardiac magnetic resonance imaging (CMR) is a powerful diagnostic modality that provides detailed quantitative assessment of cardiac anatomy and function. Automated extraction of CMR measurements from clinical reports that are typically stored as unstructured text in electronic health record systems would facilitate their use in research. Existing machine learning approaches either rely on large quantities of expert annotation or require the development of engineered rules that are time-consuming and are specific to the setting in which they were developed.

**Objective:**

We hypothesize that the use of pretrained transformer-based language models may enable label-efficient numerical extraction from clinical text without the need for heuristics or large quantities of expert annotations. Here, we fine-tuned pretrained transformer-based language models on a small quantity of CMR annotations to extract 21 CMR measurements. We assessed the effect of clinical pretraining to reduce labeling needs and explored alternative representations of numerical inputs to improve performance.

**Methods:**

Our study sample comprised 99,252 patients that received longitudinal cardiology care in a multi-institutional health care system. There were 12,720 available CMR reports from 9280 patients. We adapted PRAnCER (Platform Enabling Rapid Annotation for Clinical Entity Recognition), an annotation tool for clinical text, to collect annotations from a study clinician on 370 reports. We experimented with 5 different representations of numerical quantities and several model weight initializations. We evaluated extraction performance using macroaveraged *F*_1_-scores across the measurements of interest. We applied the best-performing model to extract measurements from the remaining CMR reports in the study sample and evaluated established associations between selected extracted measures with clinical outcomes to demonstrate validity.

**Results:**

All combinations of weight initializations and numerical representations obtained excellent performance on the gold-standard test set, suggesting that transformer models fine-tuned on a small set of annotations can effectively extract numerical quantities. Our results further indicate that custom numerical representations did not appear to have a significant impact on extraction performance. The best-performing model achieved a macroaveraged *F*_1_-score of 0.957 across the evaluated CMR measurements (range 0.92 for the lowest-performing measure of left atrial anterior-posterior dimension to 1.0 for the highest-performing measures of left ventricular end systolic volume index and left ventricular end systolic diameter). Application of the best-performing model to the study cohort yielded 136,407 measurements from all available reports in the study sample. We observed expected associations between extracted left ventricular mass index, left ventricular ejection fraction, and right ventricular ejection fraction with clinical outcomes like atrial fibrillation, heart failure, and mortality.

**Conclusions:**

This study demonstrated that a domain-agnostic pretrained transformer model is able to effectively extract quantitative clinical measurements from diagnostic reports with a relatively small number of gold-standard annotations. The proposed workflow may serve as a roadmap for other quantitative entity extraction.

## Introduction

Cardiac magnetic resonance imaging (CMR) facilitates the characterization of many important cardiac diseases including left and right ventricular failure, left ventricular hypertrophy, and aortic root aneurysms. Quantification of left ventricular ejection fraction (LVEF) and classification of patients with heart failure into those with reduced, moderately reduced, or preserved ejection fraction is the cornerstone of selecting appropriate therapies for a given patient [1]. CMR also quantifies right ventricular function and is notably the only noninvasive diagnostic modality able to fully evaluate the right ventricle [2]. Anatomic information from CMR is also diagnostic of other important cardiac diseases, including left ventricular hypertrophy, which is an important marker for overall cardiac health, and thoracic aortic root aneurysms [3]. CMR measurements, in addition to other diagnostic information, are embedded in narrative clinical text. In many electronic health record (EHR) systems, these measurements are unavailable in easily accessible harmonized structured formats. The development of tools to automatically extract quantitative measurements from unstructured CMR reports would facilitate their use in research, including as inputs to machine learning models.

Existing approaches for extracting measurements from clinical text are often based on manually developed heuristics or machine learning methods that learn from labeled data but do not leverage pretrained language representations. Rule-based approaches [4], while computationally efficient, require substantial manual effort to construct and can suffer performance degradation with shifts in linguistic structure of reports [5]. Other work has used machine learning approaches such as support vector machines and long short-term memory models to extract measurements from clinical notes, but these approaches have required large quantities of expert annotations due to absence of pretraining [6]. In addition, prior methods for clinical measurement extraction rely on considerable data-specific preprocessing, which may not translate well to EHRs outside of where the heuristics were developed [7].

Transformer-based neural networks like Bidirectional Encoder Representations from Transformers (BERT) [8,9] have achieved state-of-the-art results across a wide variety of natural language processing (NLP) tasks [10]. These models are pretrained on large amounts of text to learn general linguistic structure and produce contextualized representations of language. The advantage of this pretraining paradigm is that these networks can be fine-tuned using minimal problem-specific labels to achieve state-of-the-art performance on many natural language tasks. BERT was originally pretrained on general domain text such as Wikipedia but has since been adapted for use in clinical applications by pretraining on domain-specific text [11-14]. Although transformer-based models have shown efficacy in extracting nonnumerical entities such as anatomical terms and disease states from clinical text [14], their application to extracting numerical quantities from clinical text has been limited [15,16].

In this study, we hypothesized that pretrained transformers fine-tuned on a small set of annotations can efficiently extract numerical quantities from diagnostic text. We fine-tuned a range of pretrained transformers, including clinically oriented ones, to develop an NLP workflow that simultaneously extracts 21 specific measurements of cardiac structure and function from CMR reports in a cardiology-based EHR cohort. This set represents all clinically meaningful quantitative imaging findings available in the CMR reports. We also explored whether alternative numerical representations impact extraction quality compared to the default representations that appear in reports. After selecting the best-performing model, we applied our workflow to extract measurements from all available CMR reports in the study cohort. To demonstrate the accuracy of these extractions, we assessed the expected associations between extracted cardiac anatomy and function indices and incident clinical outcomes.

## Methods

### Study Sample

**Figure 1:**
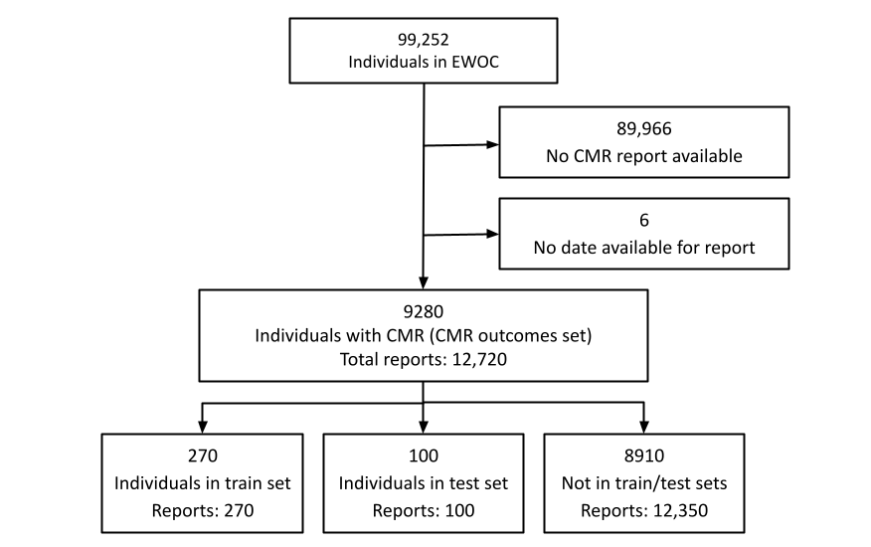
CONSORT (Consolidated Standards of Reporting Trials) diagram for study sample. CMR: cardiac magnetic resonance imaging; EWOC: Enterprise Warehouse of Cardiology.

Individuals were selected from a retrospective community-based ambulatory cardiology sample (Enterprise Warehouse of Cardiology [EWOC]) in a multi-institutional academic health care system (Mass General Brigham). EWOC comprises 99,252 adults aged 18 years or older with ≥2 cardiology clinic visits within 1 to 3 years between 2000 and 2019. A broad range of EHR data are available for each individual in the cohort, including demographics, anthropometrics, vital signs, narrative notes, laboratory results, medication lists, radiology and cardiology diagnostic test results, pathology reports, and procedural and diagnostic administrative billing codes [16]. These data were processed using the JEDI Extractive Data Infrastructure [17]. After excluding 6 individuals and reports that had no CMR date available, 12,720 CMR reports were available for 9280 individuals in EWOC (Figure 1).

### Ethics Approval

This research was approved by the Massachusetts General Brigham Institutional Review Board (2017P001650).

### Clinical Feature Ascertainment

Baseline characteristics were defined using previously published groupings of International Classification of Diseases, 9th and 10th revision diagnosis codes [16]. Definitions for clinical features used in the analysis are provided in Table S1 in Multimedia Appendix 1. Baseline characteristics of individuals in the modeling sample were ascertained prior to the date of the CMR (Table 1).

**Table 1:** Baseline characteristics of training the set, test set, and CMR outcomes set.

			Training set (N=278)	Test set (N=100)		CMR^a^ outcomes set^b^ (N=9280)
Age (years), median (Q1, Q3)			54 (46, 64)	58 (45, 66)		57 (46, 67)
Female sex, n (%)			95 (34.2)	33 (33)		3666 (39.5)
Diabetes mellitus, n (%)			23 (8.3)	10 (10)		1216 (13.1)
Coronary artery disease, n (%)			69 (24.8)	31 (31)		3406 (36.7)
Myocardial infarction, n (%)			42 (15.1)	15 (15)		1791 (19.3)
Atrial fibrillation, n (%)			104 (37.4)	24 (24)		3164 (34.1)
Obesity, n (%)			12 (4.3)	7 (7)		631 (6.8)
Chronic kidney disease, n (%)			26 (9.4)	7 (7)		1123 (12.1)
Hypertension, n (%)			130 (46.8)	55 (55)		5563 (59.9)
**Ethnicity, n (%)**						
	White	237 (85.3)		93 (93)	7814 (84.2)	
	Asian	14 (5.0)		1 (1)	251 (2.7)	
	Black	13 (4.7)		2 (2)	520 (5.6)	
	Other	7 (2.5)		1 (1)	195 (2.1)	
	Hispanic	4 (1.4)		0 (0)	111 (1.2)	
	Unknown	3 (1.1)		3 (3)	390 (4.2)	

^a^CMR: cardiac magnetic resonance imaging.

^b^Includes all individuals in Enterprise Warehouse of Cardiology with a CMR report.

### CMR Labeling

**Figure 2:**
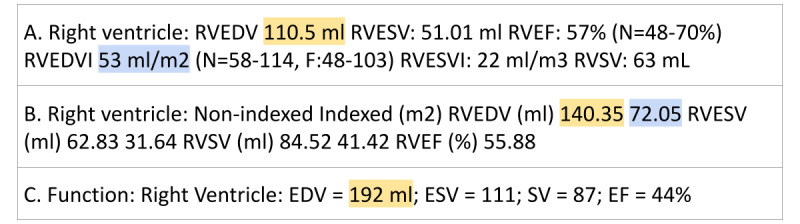
Example text from 3 cardiac magnetic resonance imaging reports (A,B,C) quantifying right ventricular function. The lack of consistency in how equivalent measurements are presented makes accurately extracting measurements challenging. Yellow highlighted features indicate right ventricular end diastolic volume (RVEDV), whereas blue highlighted features indicate right ventricular end diastolic volume index (RVEDVI). Example C does not contain the RVEDVI feature. EDV: end diastolic volume; EF: ejection fraction; ESV; end systolic volume; RVEF: right ventricular ejection fraction; RVESV: right ventricular end systolic volume; RVESVI: right ventricular end systolic volume index; RVSV: right ventricular stroke volume.

Similar to other EHRs, quantitative CMR measurements are contained in free-text diagnostic reports in the Mass General Brigham EHR [14,18]. We leveraged PRAnCER (Platform Enabling Rapid Annotation for Clinical Entity Recognition) [19], an open-source software application for intuitive labeling, to annotate 21 clinically important measurements from EWOC CMR reports (Textbox 1). We adapted PRAnCER to work with a custom schema containing CMR features rather than the Unified Medical Language System vocabulary [20] for which it was designed. There is significant variability in the format and context of measurement instances. This includes the ordering of measurements in the report, the language used to reference a particular measurement, the presence or absence of units, and the positional relationship between a measurement name and the value itself (Figure 2).

Of all available reports, 370 were randomly selected from unique individuals for annotation by a study clinician (JSH). From these reports, 270 were randomly partitioned into a training set while the remaining 100 were reserved for model testing (Figure 1). No individuals appeared in both the training and test sets. As CMR protocols may vary based on the clinical indication for the study, the total number of measurements per report ranged from 1 to 21. The counts of each unique feature across the training and test sets are available in Table S2 in Multimedia Appendix 1. Total clinician labeling time for all 370 reports was estimated at 15 hours.

Finally, to address the quality of clinical annotations, we employed a secondary annotator (PB) to label only the 100 reports reserved for model testing. We computed interannotator agreement as the proportion of matched extractions between annotators, in line with clinical entity extraction literature [15]. Overall agreement was excellent at 91.6%, and measurementwise agreement values are available in Table S3 in Multimedia Appendix 1**.** Given the nature of the annotation task, there was perfect precision when both annotators picked out a measurement from a report, and any disagreement represents values missed due to fatigue or difference in guidelines. Given the high agreement, we performed model derivation and validation on annotations from the study clinician (JSH) only.

**Clinical measurements extracted from cardiac magnetic resonance imaging reports.**

**Left ventricle anatomy and function**

- Left ventricular end diastolic volume
- Left ventricular end diastolic volume index
- Left ventricular end diastolic diameter
- Left ventricular end systolic volume
- Left ventricular end systolic volume index
- Left ventricular end systolic diameter
- Left ventricular ejection fraction
- Left ventricular stroke volume
- Left ventricular mass
- Left ventricular mass index
- Cardiac output
- Cardiac index

**Right ventricle anatomy and function**

- Right ventricular end diastolic volume
- Right ventricular end diastolic volume index
- Right ventricular end systolic volume
- Right ventricular end systolic volume index
- Right ventricular stroke volume
- Right ventricular stroke volume

**Other cardiac structural anatomy**

- Left atrial anterior-posterior dimension
- Pulmonary artery dimension
- Aortic root dimension

### Numerical Representations

Previous work has shown that the use of alternative representations in place of default surface representations of numbers has a significant impact on a transformer model’s ability to perform quantitative manipulations within text, such as simple arithmetic [21]. The vocabularies of most transformer-based models include a limited number of numerical values and generally no decimal numbers since they are constructed from the most frequently occurring words in the corpus used for pretraining. The tokenization procedure employed by most transformer models separates “words” based on punctuation and does not distinguish between periods and decimal places, which results in decimal numbers being broken up into multiple tokens. Given the potential limitations of default numerical representations, we investigated whether implementing alternative numerical representations impacts the extraction quality of quantitative clinical measures. We designed 4 different types of numerical transformations for quantitative tokens in the CMR reports, which were applied to both the training and test samples for model derivation. These included replacing decimal points with a special token to ensure that decimal numbers stay intact during tokenization, a consistent number of digits for all values, scientific notation, and converting quantities to words. Table 2 demonstrates these transformations for 1 snippet of text, and Multimedia Appendix 1 contains more information about their implementations.

**Table 2:** Numerical transformations for an example snippet of text.

Transformation name	Transformed snippet	Notes
Original	RVESV^a^: 51.01 ml	No transformation; for reference
Replaced decimal	RVESV: 51|01 ml	Decimal points replaced with special separator character; enables parsing as a single token rather than being broken up
Consistent digits	RVESV: 051010 ml	All numbers converted to be 6 digits in length
Scientific notation	RVESV: 5.10100e+01	All numbers converted to scientific notation, with 5 significant digits
Words	RVESV: fifty one point zero one ml	Number converted to corresponding word representation

^a^RVESV: right ventricular end systolic volume.

### Model Derivation and Validation

**Figure 3:**
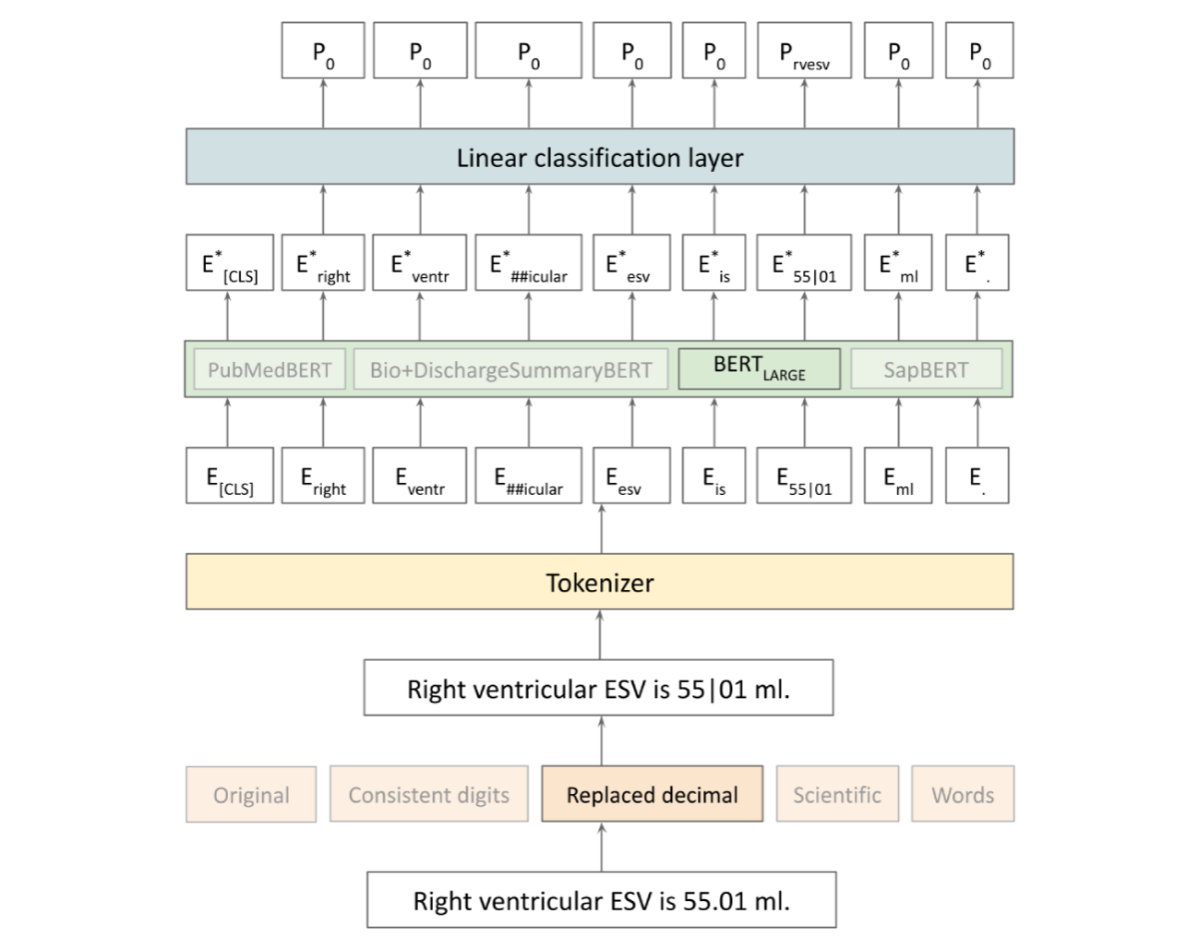
Architecture for fine-tuning pretrained transformer architecture with gold-standard cardiac resonance imaging annotations and predicting labels for each token. BERT: Bidirectional Encoder Representations from Transformers; ESV: end systolic volume.

Our modeling approach involved fine-tuning transformer-based models using the HuggingFace transformers library [22] to predict a label for each token in a given CMR report. To do so, we attached a linear classification head on top of the last layer of a BERT architecture. The classification head produces a distribution over 22 possible labels—the 21 cardiac measurements of interest plus a “0” label for all other tokens (Figure 3). We preprocessed report text into sections containing 128 tokens, accounting for subword tokenization, in accordance with input size limitations of the transformer-based models. We used cross-entropy loss with a learning rate of 5e^–5^ and a batch size of 32 across all experiments. To evaluate the impact of clinical pretraining on numerical clinical value extraction, we experimented with initializing the weights of the BERT architecture with the weights provided by BERT_LARGE_ [8,9] cased (~340 million parameters) as well as the clinically oriented weights of PubMedBERT [11], SapBERT [12], and Bio+DischargeSummaryBERT [13] (each with ~110 million parameters). Pretrained weights were downloaded from the HuggingFace model hub [23]. Each pretrained architecture was paired with the 5 numerical representations.

Each model was fine-tuned on the Center for Clinical Data Science computational cluster hosted by Mass General Brigham. On a graphic processing unit–equipped machine, each model trained at a rate of approximately 2 minutes per epoch. Each combination of weight initialization and numerical representation strategy was fine-tuned for 20 epochs, requiring an average of 40 minutes. For the purpose of model evaluation, we assigned a label to a token if the predicted score for that label was greater than 0.5. Performance was evaluated using the macroaveraged *F*_1_-score over all 21 measurements of interest, as this metric captures featurewise performance regardless of the frequency of occurrence in the reports. For each model, we selected the number of epochs that maximized the macroaveraged *F*_1_-score.

**Figure 4:**
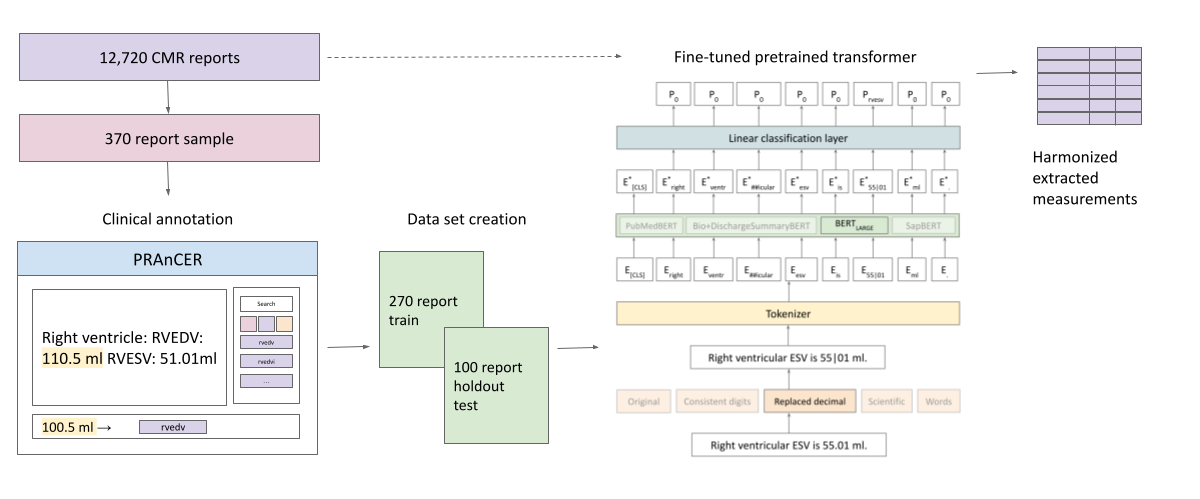
Natural language processing workflow for collecting clinical annotations, modeling, and extracting measurements from cardiac magnetic resonance imaging reports. BERT: Bidirectional Encoder Representations from Transformers; ESV: end systolic volume; CMR: cardiac magnetic resonance imaging; PRAnCER: Platform Enabling Rapid Annotation for Clinical Entity Recognition; RVEDV: right ventricular end diastolic volume; RVESV: right ventricular end systolic volume.

Minimal postprocessing was applied based on the results of the labels assigned by our modeling experiments. This included merging with additional significant digits that should obviously be included as part of a measurement and the consolidation of model-predicted tokens into a structured format (Multimedia Appendix 1)**.** Finally, we applied upper and lower bounds on extracted values using reference ranges derived from the CMR literature [24-26] (Table S4, Multimedia Appendix 1). An overview of the workflow, including collecting clinical annotations, modeling, and postprocessing to extract final measurements is provided in Figure 4.

### Associations With Clinical Outcomes

Finally, to assess the clinical validity of model extractions, we evaluated whether selected extracted features demonstrated known relationships with clinical outcomes, including mortality, atrial fibrillation, and heart failure [27-29]. We first applied the highest performing model to extract left ventricular mass index (LVMI), LVEF, and right ventricular ejection fraction (RVEF) from all CMR reports in EWOC. Rather than choose a model score threshold for each label, we chose the label with the highest score for each token. For individuals with multiple reports containing a given feature, we used features extracted from the earliest report for the primary analysis.

We then assessed incidence rates of mortality, atrial fibrillation, and heart failure by quartile of extracted left ventricular mass. We also measured the incidence rate of mortality by abnormal and normal LVEF and RVEF, defined as LVEF <50% and RVEF <45%, respectively [1,30]. Clinical outcomes were defined using previously described groupings of diagnostic codes [31,32]. For incidence analysis, we omitted individuals with the primary outcome (ie, atrial fibrillation or heart failure) occurring prior to or on the same day as the CMR. For incident atrial fibrillation and heart failure analyses, follow-up time began at the time of the CMR and continued until occurrence of the primary outcome, death, or last clinical encounter. For mortality analysis, follow-up time began at the time of the CMR and continued until time of death or last clinical encounter. Confidence intervals were calculated by the exact method. We compared incidence rates using the 2-sample test of proportions [33]. In order to assess potential confounding of report timing on associations between extracted features and clinical outcomes, we also performed a sensitivity analysis where we selected features extracted from the last report.

## Results

### Model Performance

The training set included reports from 270 individuals with a median age of 65 (IQR 54-74) years at time of CMR of whom 34.2% (n=92) were female (Table 2). The test set included reports from 100 individuals with a median age of 58 (IQR 45-66) years at time of CMR of whom 33% (n=33) were female (Table 2).

**Figure 5:**
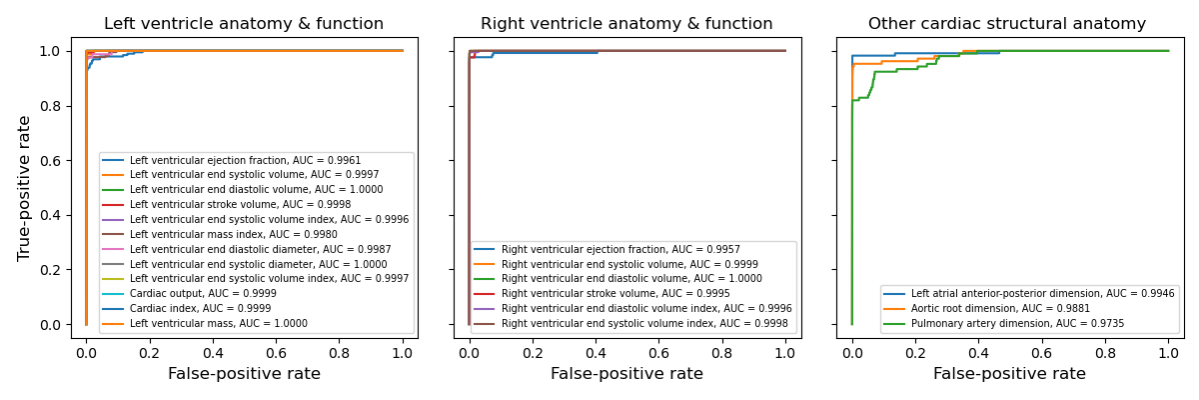
Receiver operating characteristic curves for model predictions on the test set by cardiac magnetic resonance imaging measurement. AUC: area under the receiver operating characteristic curve.

**Figure 6:**
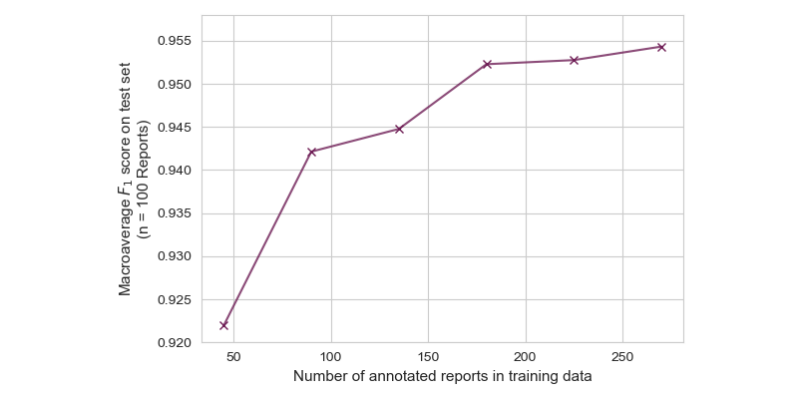
Fine-tuned BERT_LARGE_ performance with replaced decimal numerical representations, as a function of number of annotated reports in the training set.

All combinations of pretrained weights and numerical representations achieved excellent macroaveraged *F*_1_-scores on the test set. Table 3 illustrates the maximum macroaveraged *F*_1_-scores for all combinations of pretrained weight initializations and numerical representations. The best-performing combination was BERT_LARGE_, fine-tuned on the replaced decimal numerical representation scheme, which achieved a maximum macroaveraged *F*_1_-score of 0.957 after fine-tuning for 12 epochs. A plot of macroaveraged *F*_1_-score on the test set over the training epochs is available in Figure S1 in Multimedia Appendix 1**,** and featurewise receiver operating characteristic curves are shown in Figure 5. The range of feature-level macroaveraged *F*_1_-scores was 0.902 to 1.000, and all scores are reported in Table S5, Multimedia Appendix 1. To investigate the impact of labeling effort on model performance, we fine-tuned this combination of BERT_LARGE_ pretraining and the replaced decimal numerical representation scheme on varying subsets of the training data, and plotted the macroaveraged *F*_1_-score on the test set (Figure 6). This plot demonstrates consistently significant gains in performance when the number of training reports is iteratively increased from 45 to about 200 but starts to saturate after this point. We also correlated the number of annotations in the training sample with test *F*_1_ performance for each measurement and did not find a strong relationship (Figure S2, Multimedia Appendix 1).

**Table 3:** Maximum macroaveraged *F*_1_-scores and bootstrapped 95% CIs on gold-standard test labels by pretrained weight initialization and numerical representation.

Architecture	Numerical representation, maximum macroaveraged *F*_1_-score (95% CI)				
	Original	Replaced decimal	Consistent digits	Scientific	Words
PubMedBERT^a^	0.954 (0.947-0.960)	0.952 (0.947-0.960)	0.950 (0.945-0.955)	0.955^b^ (0.948-0.960)	0.953 (0.949-0.958)
SapBERT	0.955 (0.949-0.960)	0.954 (0.949-0.960)	0.955 (0.949-0.960)	0.955 (0.948-0.960)	0.956^b^ (0.951-0.961)
Bio+Discharge SummaryBERT	0.950 (0.944-0.957)	0.953^b^ (0.947-0.959)	0.953 (0.945-0.958)	0.952 (0.945-0.958)	0.946 (0.942-0.952)
BERT_LARGE_	0.951 (0.945-0.957)	0.957^b^ (0.951-0.962)	0.951 (0.945-0.957)	0.944 (0.938-0.951)	0.952 (0.947-0.957)

^a^BERT: Bidirectional Encoder Representations from Transformers.

^b^Best-performing numerical representation for each pretrained weight initialization.

In EWOC, there were 12,720 CMR reports from 9280 individuals, which composed the CMR outcomes set (Figure 1). The median age of individuals in the outcomes set at the time of CMR was 57 (IQR 46-67) years, and 39.50% (3666/9280) were female (Table 1). After selecting the best model configuration, we applied the top-performing model to infer CMR values on all reports in this set. After running inference, we filtered by physiologic lower and upper bounds (Table S6, Multimedia Appendix 1) and extracted a total of 136,407 measurements. Counts for each extracted feature and distribution metrics are illustrated in Table S7 in Multimedia Appendix 1. We also compared the proportion of reports that contained model-predicted measurements in the CMR outcomes set and found them to be consistent with gold-standard annotation proportions in the test set (Table S8, Multimedia Appendix 1).

### Associations With Clinical Outcomes

**Figure 7:**
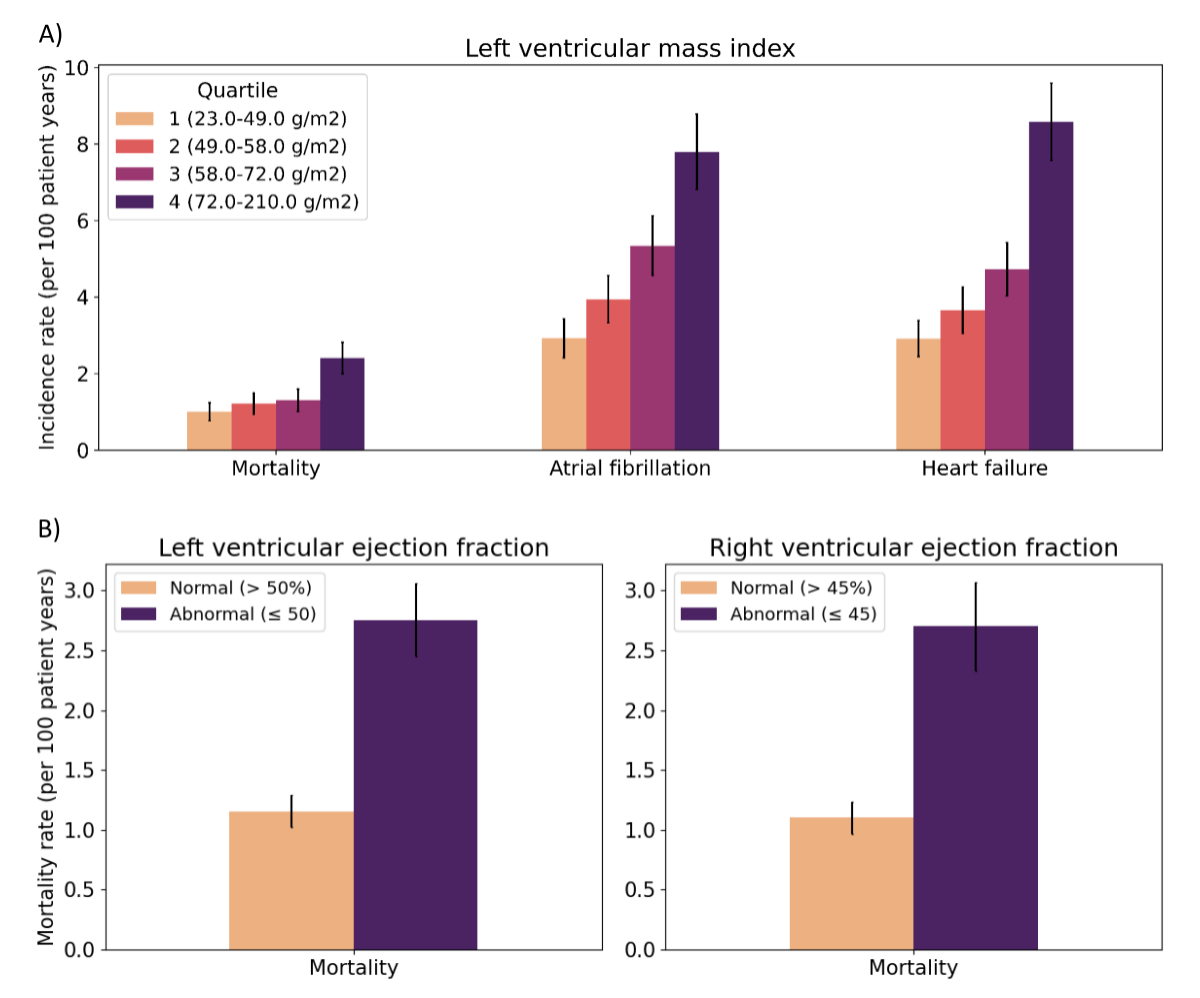
Association of extracted left ventricular mass index, left ventricular ejection fraction, and right ventricular ejection fraction with clinical outcomes.

The median follow-up time of individuals in the CMR outcomes set was 5.3 (IQR 2.8-9.2). In the outcomes set, we observed 1520 incident heart failure events, 1488 incident atrial fibrillation events, and 909 deaths during follow-up. LVMI was extracted from 5015 of 9280 individuals (54.04%). In the outcomes set, increasing LVMI was associated with increasing incidence of mortality, atrial fibrillation, and heart failure with statistically significant differences in incidence rates between the lowest and highest quartiles (Figure 7). The mortality rate was 0.9 deaths per 100 person-years (PY; 95% CI 0.7-1.1) in the lowest quartile of extracted LVMI compared to 2.2 deaths per 100 PY (95% CI 1.9-2.6) in the highest quartile of extracted LVMI (*P*<.05; Figure 7). The incidence rate of atrial fibrillation was 3.0 events per 100 PY (95% CI2.5-3.5) in the lowest quartile of extracted LVMI compared to 7.9 events per 100 PY (95% CI 6.8-8.7) in the highest quartile of extracted LVMI (*P*<.05). The incidence rate of heart failure was 3.2 events per 100 PY (95% CI 2.7-3.7) in the lowest quartile of extracted LVMI compared to 8.1 events per 100 PY (95% CI 7.2-9.1) in the highest quartile of extracted LVMI (*P*<.05).

LVEF was extracted from 7389 of 9280 individuals (79.62%), and 2297 met the criteria for abnormal LV systolic dysfunction (LVEF <50%). RVEF was extracted from 6324 of 9280 individuals (68.15%), and 1626 met criteria for abnormal RV systolic function (RVEF <45%; Figure 7). Both abnormal LVEF and RVEF were significantly associated with increased incidence of mortality compared to normal ventricular function (*P*<.05 for both measures). In the abnormal LVEF group, the mortality rate was 2.5 deaths per 100 PY (95% CI 2.2-2.8) compared to 1.1 deaths per 100 PY (95% CI 0.9-1.2) in the normal LVEF group (*P*<.05). In the abnormal RVEF group, the mortality rate was 2.5 deaths per 100 PY (95% CI 2.1-2.8) compared to 1.0 deaths per 100 PY (95% CI 0.9-1.2) in the normal RVEF group (*P*<.05).

We also performed a sensitivity analysis where the last CMR report was used for feature extraction of LVMI, LVEF, and RVEF. There were 687 of 5015 (13.70%) individuals with more than 1 extracted LVMI, 1268 of 7389 (17.16%) individuals with more than 1 extracted LVEF, and 1038 of 6324 (16.41%) individuals with more than 1 extracted RVEF. The mean time difference between the first and last reports for LVMI was 2.4 (SD 2.2) years, the LVEF was 2.9 (SD 2.9) years, and the RVEF was 2.7 (SD 2.6) years. Similar to the primary analysis, we observed increasing rates of mortality, atrial fibrillation, and heart failure with increasing LVMI; and significantly higher mortality rates in individuals with abnormal LVEF or RVEF compared to individuals with normal LVEF or RVEF (Figure S3, Multimedia Appendix 1).

## Discussion

### Principal Results

In this study, we report the results of an accurate and practical NLP-based approach for simultaneously extracting 21 quantitative measurements from CMR reports. Our final model, which yielded a macroaveraged *F*_1_-score of 0.957, was derived from a workflow leveraging open-source frameworks for collecting gold-standard clinician labels and publicly available transformer model weights. We also highlight the clinical validity of our approach by demonstrating known associations of extracted CMR measurements with outcomes such as atrial fibrillation, heart failure, and mortality (Figure 7) [30,34].

We found that BERT_LARGE_ demonstrated excellent performance when compared to model initializations based on clinically oriented pretraining, indicating that clinical pretraining does not have a significant impact on clinical numerical value extraction (Table 3). BERT_LARGE_ is larger than the available clinically oriented models, and model complexity may play a role in comparable performance, indicating that larger clinically pretrained models represent a direction for future work. We also experimented with 4 different alternative representations of numerical measurements and found the test performance to be similar to that of the default representation (Table 3). Our findings suggest that for the particular case of extracting numerical quantities, transformer-based models do not require clinical pretraining or alternative numerical representations. Through experiments with limited training set sizes, we found that excellent performance can be achieved with fewer than 50 labeled reports. Furthermore, a training set with 175 reports was sufficient to train a model with performance that was within the 95% CI of a model trained with 270 reports (Figure 6).

Measurements extracted by our model potentially facilitate the automated characterization of a range of important cardiac diseases, which we leave to future work. We expect that our proposed workflow can be easily used by others to extract arbitrary measurements from clinical text. The PRAnCER platform is open source and can be easily adapted to label clinical measurements of interest. Our software for fine-tuning and evaluating NLP models is also open source [34], and model training is possible using a standard graphic processing unit–equipped machine. We expect it to be possible to extract an arbitrary number of clinical measurements with a practical amount of labeling effort and computational requirements in clinical domains not limited to CMRs.

### Attention-Based Exploration of Error Modes

The characterization of error modes can be instructive toward having confidence in model predictions and for finding ways to improve a model by future researchers. Despite the overall high accuracy of our best model across all the types of measurements that we considered, the most common error mode involved the model assigning a “0” label to values that should have been labeled as measurements. In many cases that we examined, a measurement such as “aortic root dimension” would be correctly labeled in one report and not labeled in another report despite a similar sequence of tokens surrounding the value to be labeled. By examining the attention weights for the token to be labeled in both reports, we discovered that the correctly labeled value most heavily weighted the word “dimension” in the preceding “aortic root dimension” phrase. For the incorrectly labeled value, 3 of the 4 most-attended tokens were separate instances of the word “dimension,” one of which was part of the correct phrase, with the other instances appearing in the remainder of the text. All of the attention weights were much lower than the attention paid to the word “dimension” by the correctly labeled example. This may indicate that an opportunity for further improvement could involve providing more training examples with sections of text that are absent from most reports in our data set or by augmenting existing labeled text with synthetic text containing critical tokens.

Additionally, we recognize that while our models perform well, extraction errors are inevitable. The clinical consequences of these errors depend on the specific feature. For example, incorrect LVEF extraction could misclassify a patient with heart failure as reduced ejection fraction or preserved ejection fraction and thereby impact treatment choices. Similarly, incorrect RVEF could misclassify a patient with right-sided heart failure. Incorrect aortic root size could misclassify an aortic root aneurysm. False-positive errors may be particularly difficult to detect as the final postprocessing stop of physiologic filtering means that false positives will still be within the expected range. Therefore, careful evaluation of model performance is necessary, especially if applying such a model to new data sets.

### Comparison With Prior Work

To our knowledge, this is the first example of using a transformer-based model (without pretraining from scratch) fine-tuned on clinician labels to extract numerical measurements from diagnostic text. We previously demonstrated the value of extracting 4 vital sign measurements from clinical text based on a large number of weak labels that were generated using a rule-based approach [16]. Our previous approach was based on the assumption that it would be impractical to accrue a sufficient quantity of gold-standard annotations in order to fine-tune a transformer-based approach. However, we found that a single clinician required at most 15 hours to produce sufficient gold-standard annotations for 21 types of quantitative measurements, thereby eliminating the need for rule-based approaches and enabling easy scaling to a large number of relevant measurements.

Recent work [15] used a combination of embeddings produced by pretraining a BERT model and a FLAIR model from scratch on domain-specific data. Embeddings were then used as input to a combination of a bidirectional long short-term memory with a conditional random field layer to label tokens of interest, including numerical measurements. This approach worked well and achieved comparable performance to our approach with a similar amount of labeling effort. We demonstrate with our work that pretraining a model from scratch on domain-specific data is not necessary to achieve a high level of accuracy. The days, or perhaps even weeks, of computation required to pretrain a model from scratch on clinical data can be avoided. Furthermore, our work examines the impact of the number of annotations on performance.

Other approaches for extracting numerical measurements from clinical text have also achieved reasonable accuracy, but we suggest that our approach minimizes labeling effort, is more robust, and is sufficiently computationally efficient to serve as a practical solution for accelerating EHR-based clinical research. Rule-based approaches, while potentially accurate, generally require multiple iterations of development and validation to ensure accuracy given the wide variability of clinical text [4]. Prior work has also shown that rule-based approaches may not be easily portable to other EHRs outside of where they were developed. In their work evaluating the portability of a rule-based model for extraction of echocardiogram measurements, Adekkanattu et al [7] report variable *F*_1_-scores that differ by clinical site. We demonstrate that transformer-based models pretrained on clinical text can be fine-tuned on a practical number of labels to learn to extract measurements in a way that is flexible to variability in how such measurements are expressed in clinical text.

### Limitations and Directions for Future Work

Our study must be interpreted in the context of its limitations. Our test set consisted of a relatively small sample of 100 reports, but an analysis to randomly resample the test set of the same size yielded models with a markedly close range of macro *F*_1_-scores (0.947-0.970 across 10 samples), which indicates the robustness of our approach. Our approach required a minimal degree of postprocessing and mainly involved imposing physiologic ranges for values extracted by the model. Although relatively few values were filtered this way, these may represent model false positives. Another aspect of postprocessing involved extending model predictions to include missed significant digits, which happened very rarely. Our experiments with numerical representations and pretrained models enabled high extraction accuracy, but further work is required to understand how to best use transformer-based models in handling arbitrary numerical values [35]. In addition, CMR reports were taken from a large heterogeneous health care system, and while our model was able to handle significant variability in the presentation of relevant measurements, further work is required to show that our modeling approach is portable to other institutions.

Similar to other artificial intelligence models with health care applications, clinical implementation of our model is stymied by several barriers [36]. The first is deployment of a model within an EHR environment, which involves both accessing siloed clinical data and integrating modeling results into the electronic environment for presentation. The second is ensuring that the model is adaptable to changes in report structure either between institutions or prospectively over the lifetime of the model. Last, monitoring and regular quality control is essential to ensuring patient safety. Although few models have successfully overcome these numerous challenges, we hypothesize that our work offers a modeling strategy that is adaptable to changes in report structure and provides a framework for developing new quantitative models aimed at other important clinical tasks. Future work should test the performance of models like these in real-time settings to prove generalizability to new environments and data structures.

### Conclusions

We present a powerful natural language workflow for simultaneously extracting 21 types of numerical measurements from CMR free-text reports. We found that general pretrained transformer-based language models require a relatively small number of gold-standard annotations, necessitate minimal data processing, and are robust to significant variability in the context and presentation of numerical measurements. We observed expected associations between extracted CMR measurements and known clinical outcomes like heart failure, atrial fibrillation, and mortality. Our workflow is reproducible and is likely applicable to many other types of clinical data.

## Appendix

Multimedia Appendix 1 Supplemental material.

## Abbreviations

BERT: Bidirectional Encoder Representations from Transformers
CMR: cardiac magnetic resonance imaging
EHR: electronic health record
EWOC: Enterprise Warehouse of Cardiology
LVEF: left ventricular ejection fraction
LVMI: left ventricular mass index
NIH: National Institutes of Health
NLP: natural language processing
PRAnCER: Platform Enabling Rapid Annotation for Clinical Entity Recognition
PY: person-years
RVEF: right ventricular ejection fraction
