# Supplementary material for: One Clinician Is All You Need–Cardiac Magnetic Resonance Imaging Measurement Extraction: Deep Learning Algorithm Development
Source: JMIR Med Inform. 2022 Sep 16;10(9):e38178. doi: 10.2196/38178 (PMC9526125; doi:10.2196/38178)
Supplement: Multimedia Appendix 1 [file medinform_v10i9e38178_app1.doc]

**MULTIMEDIA APPENDIX:**

One clinician is all you need – Cardiac Magnetic Resonance Imaging Measurement Extraction: Deep Learning Algorithm Development

This is a Multimedia Appendix to a full manuscript published in the J Med Internet Res. For full copyright and citation information see http://dx.doi.org/10.2196/jmir.xxxx

Table of Contents

[Methods I: Implementation of Numerical Transformations 3](#__RefHeading___Toc112240589)

[Methods II: Model prediction processing and consolidation 4](#__RefHeading___Toc112240590)

[Figure I. Learning curve for the best model -- BERT_LARGE_ fine-tuned on Replaced Decimal numerical representation 5](#__RefHeading___Toc112240591)

[Figure II: Investigating the Relationship between Train Annotation Count and Test F1 by Measurement Type. 6](#__RefHeading___Toc112240592)

[Figure III: Association of extracted left ventricular mass index, left ventricular ejection fraction, and right ventricular ejection fraction with clinical outcomes using last cardiac MRI report for feature extraction 7](#__RefHeading___Toc112240593)

[Table I. Clinical factor definitions 8](#__RefHeading___Toc112240594)

[Table II: List of clinical measurements extracted, associated counts of gold-standard clinician annotations 10](#__RefHeading___Toc112240595)

[Table III: Inter-Annotator Agreement Across Measurements in the Test Sample (n=100 Reports) 11](#__RefHeading___Toc112240596)

[Table IV: Upper and Lower Bounds to Filter Model Extractions 12](#__RefHeading___Toc112240597)

[Table V: Feature-level performance for BERT_LARGE_, fine-tuned for 12 epochs, evaluated on 100 reports annotated with gold-standard clinician labels. 13](#__RefHeading___Toc112240598)

[Table VI: Results of filtering model extractions by physiologic lower- and upper- bounds 14](#__RefHeading___Toc112240599)

[Table VII: Counts and distribution metrics for model extracted measurements across the study cohort 15](#__RefHeading___Toc112240600)

[Table VIII: Model-extracted measurement occurrence across reports in study cohort, in reference to gold standard annotations 16](#__RefHeading___Toc112240601)

## **Methods I:** Implementation of Numerical Transformations

We investigated the impact of surface numerical representations on the ability of pre-trained transformers to extract numerical quantities embedded in narrative clinical text. In addition to leaving the measurements in the original form in which they appeared, we implemented four additional numerical transformations, as illustrated in Table 2 of the main manuscript. These transformations were applied. The implementation details for each are described below:

- **Replaced Decimal**: For every numerical token that contained a decimal point, we replaced it with the pipe character. Whole numbers were left unchanged. So, the token ‘2.0’ would be represented as ‘2|0’, while the token ‘2’ would remain consistent. This substitution of decimals enables numerical quantities to be parsed as a single token rather than being broken up into multiple sub-words during the standard tokenization process.
- **Consistent Digits:** Every numerical token, regardless of whether it contained a decimal point, was converted to a representation containing six digits. This approach also enables each quantity to be represented as a single token with standard tokenization, with the added benefit of consistent length, which the model might be able to learn to incorporate into its predictions. This conversion is done by taking the integer-part of the quantity and prepending an appropriate number of zeros to obtain three digits, and correspondingly taking the fractional part of the quantity and appending an appropriate number of zeros to also obtain three digits. This results in a six digit representation, where a decimal point placed after the third digit would effectively reverse the transformation. With this approach, the number ‘1.667’ would be represented as ‘001667’, while the number ‘105.1’ would be transformed to ‘105100’.
- **Scientific Notation:** Each number is converted to scientific notation using Python’s native string formatting capabilities, with five significant digits after the decimal place [[1]](https://paperpile.com/c/80qaDl/FfQEa). The number ‘105.1’ would thus be transformed to ‘1.05100e+01’.
- **Words:** Each number is converted to its corresponding cardinal description using the num2words library [[2]](https://paperpile.com/c/80qaDl/SDDhR), and any ‘-’ tokens are replaced with whitespace. Thus, the number ‘105.1’ would be converted to the phrase ‘one hundred and five point one’.

## **Methods II:** Model prediction processing and consolidation

#

Minimal post-processing was applied to extract numerical values given the labels predicted by one of our trained models for a given cMR report. The model predicts a label for each token in the input text. These labels correspond to one of the measurements of interest plus a 0 for all other tokens. Series of contiguous tokens assigned the same non-0 label by the model were merged into one token with that label. When tokens following a token with a non-0 label appeared to be digits that should have been assigned the same label by the model they were also merged with the preceding labeled token.

Most measurements of interests have standard units that are used across reports. For measurements corresponding to length (left ventricular end systolic diameter, left ventricular end diastolic diameter, left atrial anterior-posterior dimension, aortic root dimension, pulmonary artery dimension), measurements appeared either in centimeters or millimeters. For these sets of measurements, we looked for unit tokens immediately before and after model predictions, and used these to standardize the units of all instances. Finally, we stored all extracted model measurements in structured format, with each row in the tabular format containing information about the extracted measurement, the report it was extracted from, and metadata for the individual, enabling the use of these cMR measurements for research applications.

## **Figure I.** Learning curve for the best model -- BERT_LARGE_ fine-tuned on Replaced Decimal numerical representation

**
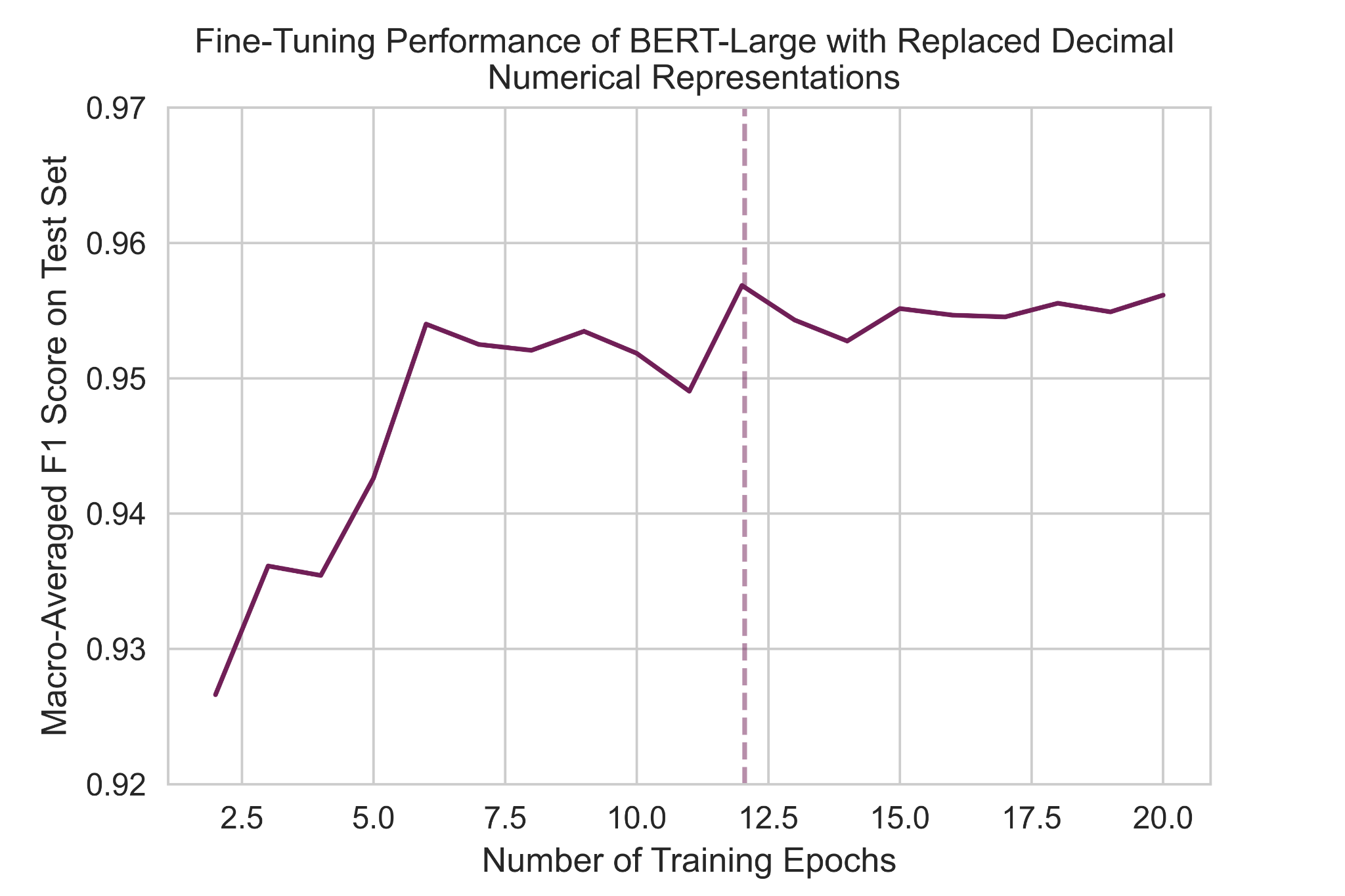
**

## **Figure II:** Investigating the Relationship between Train Annotation Count and Test F1 by Measurement Type.

**
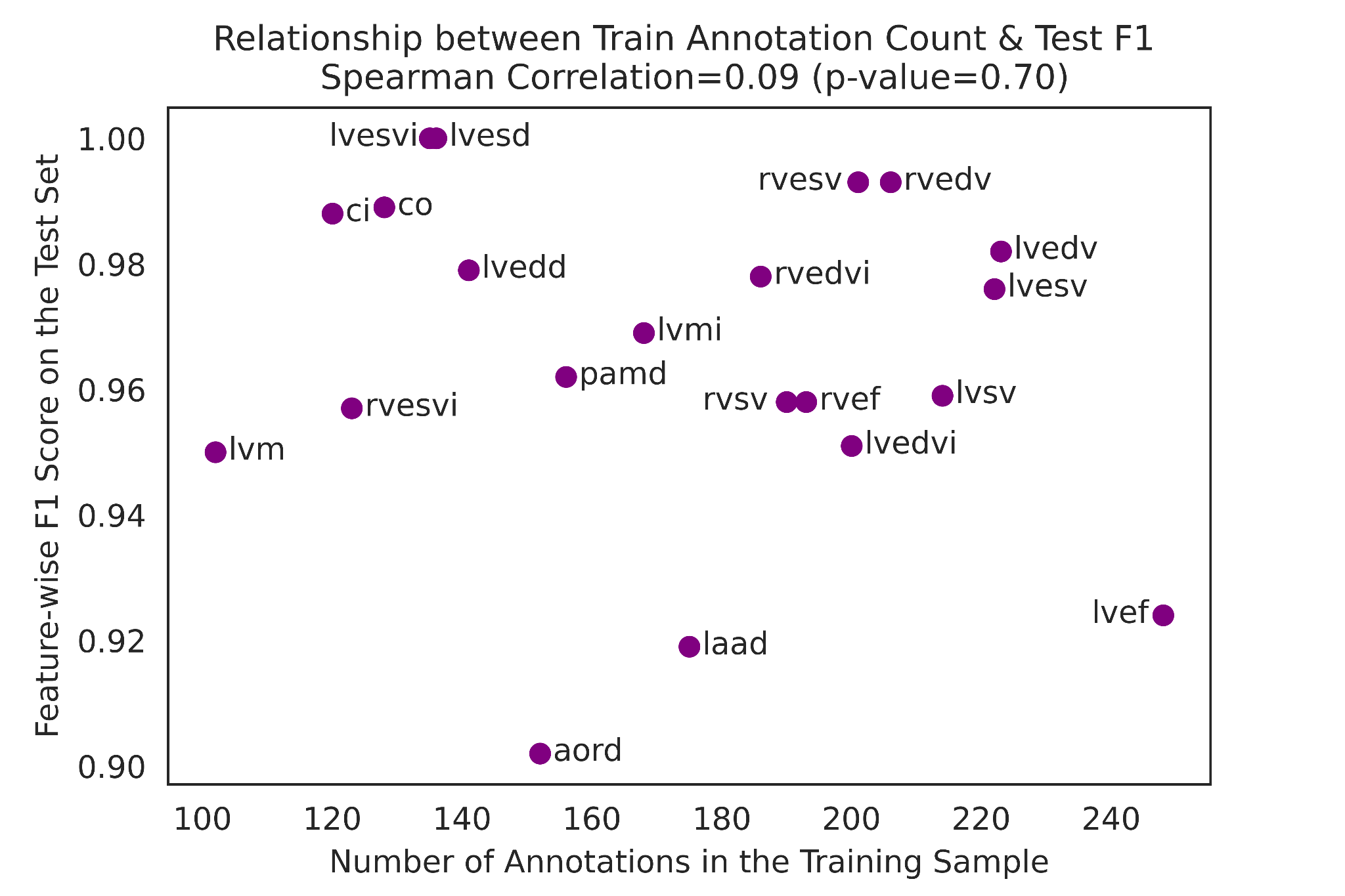
**

## **Figure III**: Association of extracted left ventricular mass index, left ventricular ejection fraction, and right ventricular ejection fraction with clinical outcomes using last cardiac MRI report for feature extraction


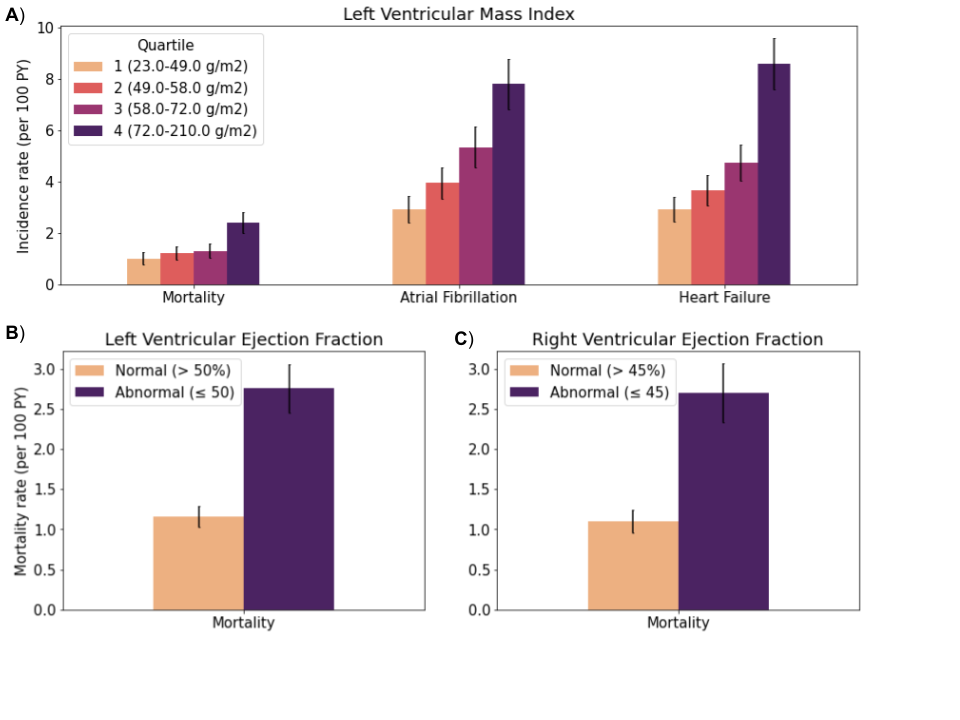


Association of A) extracted left ventricular mass index with mortality, atrial fibrillation, and heart failure by quartile, B) left ventricular ejection fraction with mortality by normal (> 50%) versus abnormal (< 50%), and C) right ventricular ejection fraction with mortality by normal (> 45%) versus abnormal (< 45%). Error bars represent 95% confidence intervals.

#

## **Table I.** Clinical factor definitions

| Phenotype | Code type | Data codes |
| --- | --- | --- |
| CKD | ICD-9 | 250.4, 250.41, 250.42, 250.43, 403, 403.01, 403.1, 403.11, 403.9, 403.91, 404, 404.01, 404.02, 404.03, 404.1, 404.11, 404.12, 404.13, 404.9, 404.91, 404.92, 404.93, 582, 582.1, 582.2, 582.4, 582.81, 582.89, 582.9, 583, 583.1, 583.2, 583.4, 583.6, 583.7, 583.81, 583.89, 583.9, 584.5, 584.6, 584.7, 584.8, 584.9, 585.1, 585.2, 585.3, 586, 587, 588, 588.81, 588.89, 588.9, 753, 753.12, 753.13, 753.14, 753.15, 753.16, 753.17, 753.19, 788.5, 792.5 |
| CKD | ICD10 | E08.22, E09.22, E10.22, E11.22, E13.22, I12.0, I12.9, I13.0, I13.1, I13.10, I13.11, I13.2, N18.1, N18.2, N18.3, N18.4, N18.5, N18.9, N19, N99.0, R34 |
| Diabetes | ICD9 | 249, 249.01, 249.1, 249.11, 249.2, 249.21, 249.3, 249.31, 249.4, 249.41, 249.5, 249.51, 249.6, 249.61, 249.7, 249.71, 249.8, 249.81, 249.9, 249.91, 250, 250.01, 250.02, 250.03, 250.1, 250.11, 250.12, 250.13, 250.2, 250.21, 250.22, 250.23, 250.3, 250.31, 250.32, 250.33, 250.4, 250.41, 250.42, 250.43, 250.5, 250.51, 250.52, 250.53, 250.6, 250.61, 250.62, 250.63, 250.7, 250.71, 250.72, 250.73, 250.8, 250.81, 250.82, 250.83, 250.9, 250.91, 250.92, 250.93, 357.2, 362.01, 362.02, 362.03, 362.04, 362.05, 362.06, 362.07, 366.41, 791.6 |
|  |  | E08.00, E08.01, E08.10, E08.11, E08.21, E08.22, E08.29, E08.311, E08.319, E08.321, E08.329, E08.331, E08.339, E08.341, E08.349, E08.351, E08.359, E08.36, E08.39, E08.40, E08.41, E08.42, E08.43, E08.44, E08.49, E08.51, E08.52, E08.610, E08.618, E08.620, E08.621, E08.622, E08.628, E08.630, E08.638, E08.641, E08.649, E08.65, E08.69, E08.8, E08.9, E08.90, E09.00, E09.01, E09.10, E09.11, E09.21, E09.22, E09.29, E09.311, E09.319, E09.321, E09.329, E09.331, E09.339, E09.341, E09.349, E09.351, E09.359, E09.36, E09.39, E09.40, E09.41, E09.42, E09.43, E09.44, E09.49, E09.51, E09.52, E09.59, E09.610, E09.618, E09.620, E09.621, E09.622, E09.628, E09.630, E09.638, E09.641, E09.649, E09.65, E09.69, E09.8, E09.9, E10.10, E10.11, E10.21, E10.22, E10.29, E10.311, E10.319, E10.321, E10.329, E10.331, E10.339, E10.341, E10.349, E10.351, E10.359, E10.36, E10.39, E10.40, E10.41, E10.42, E10.43, E10.44, E10.49, E10.51, E10.52, E10.59, E10.610, E10.618, E10.620, E10.621, E10.622, E10.628, E10.630, E10.638, E10.641, E10.649, E10.65, E10.69, E10.8, E10.9, E11.00, E11.01, E11.21, E11.22, E11.29, E11.311, E11.319, E11.321, E11.329, E11.331, E11.339, E11.341, E11.349, E11.351, E11.359, E11.36, E11.39, E11.40, E11.41, E11.42, E11.51, E11.52, E11.59, E11.610, E11.618, E11.620, E11.621, E11.622, E11.628, E11.630, E11.638, E11.641, E11.649, E11.65, E11.69, E11.8, E11.9, E13.00, E13.01, E13.10, E13.11, E13.21, E13.22, E13.29, E13.311, E13.319, E13.321, E13.329, E13.331, E13.339, E13.341, E13.349, E13.351, E13.359, E13.36, E13.39, E13.40, E13.41, E13.42, E13.43, E13.44, E13.49, E13.51, E13.52, E13.59, E13.610, E13.618, E13.620, E13.621, E13.622, E13.628, E13.630, E13.638, E13.641, E13.649, E13.65, E13.69, E13.8, E13.9, R82.4 |
| Heart Failure | ICD-9 | 398.91, 402.01, 402.11, 402.91, 404.01, 404.03, 404.11, 404.13, 404.91, 404.93, 428, 428.1, 428.2, 428.21, 428.22, 428.23, 428.3, 428.31, 428.32, 428.33, 428.4, 428.41, 428.42, 428.43, 428.9 |
| Heart Failure | ICD-10 | I09.81, I11.0, I13.0, I13.2, I50.1, I50.20, I50.21, I50.22, I50.23, I50.30, I50.31, I50.32, I50.33, I50.40, I50.41, I50.42, I50.43, I50.9, I97.130, I97.131 |
| Coronary Artery Disease | ICD-9 | 411, 411.1, 411.81, 411.89, 413, 413.9, 414, 414.01, 414.02, 414.03, 414.04, 414.05, 414.06, 414.07, 414.1, 414.11, 414.19, 414.2, 414.3, 414.4, 414.8, 414.9, 429.2, 996.03, V45.81, V45.82 |
|  | ICD-10 | I20.0, I20.1, I20.8, I20.9, I21.01, I21.02, I21.09, I21.11, I21.19, I21.21, I21.29, I21.3, I21.4, I22.0, I22.1, I22.2, I22.8, I22.9, I23.0, I23.1, I23.2, I23.3, I23.4, I23.5, I23.6, I23.7, I23.8, I24.0, I24.8, I25.10, I25.110, I25.111, I25.118, I25.119, I25.3, I25.41, I25.42, I25.6, I25.700, I25.701, I25.708, I25.709, I25.710, I25.711, I25.718, I25.719, I25.720, I25.721, I25.728, I25.729, I25.730, I25.731, I25.738, I25.739, I25.790, I25.791, I25.798, I25.799, I25.810, I25.811, I25.812, I25.82, I25.83, I25.84, I25.89, I25.9, Z95.1, Z98.61 |
| Hypertension | ICD-9 | 401, 401.1, 401.9, 402, 402.01, 402.1, 402.11, 402.9, 402.91, 403, 403.01, 403.1, 403.11, 403.9, 403.91, 404, 404.01, 404.02, 404.03, 404.1, 404.11, 404.12, 404.13, 404.9, 404.91, 404.92, 404.93, 405.01, 405.09, 405.11, 405.19, 405.91, 405.99, 437.2, 796.2 |
|  | ICD-10 | I10, I11.0, I11.9, I12.0, I12.9, I13.0, I13.10, I13.11, I13.2, I15.0, I15.1, I15.2, I15.8, I15.9 |
| Valvular Disease | ICD-9 | 35.05, 35.06, 35.1, 35.11, 35.12, 35.13, 35.14, 35.2, 35.21, 35.22, 35.23, 35.24, 35.25, 35.26, 35.27, 35.28, 35.96, 394, 394.1, 394.2, 394.9, 396, 396.1, 396.2, 396.3, 396.8, 396.9, V42.2, V43.3 |
|  | ICD-10 | I05.0, I05.1, I05.2, I05.8, I05.9, I06.8 , I06.9, I07.8, I07.9, I08.0, I08.1, I08.3, I08.8, I08.9, I09.1, I34.0, I34.1, I34.2, I34.8, I34.9, I35.0, I35.1, I35.2, I35.8, I35.9, I36.0, I36.1, I36.2, I36.8, I36.9, I37.0, I37.1, I37.2, I37.8, I37.9, I38 |
|  | CPT | 33400, 33401, 33403, 33405, 33406, 33411, 33412, 33413, 33420, 33422, 33425, 33426, 33427, 33430, 33606, 33611, 33612, 33645, 33665, 33670, 33681, 33684, 33688, 33690 |
| Atrial fibrillation (AF) defined as ECG diagnosis of AF, ≥1 inpatient diagnosis code, ≥1 procedural code, or ≥2 codes of any type  Heart failure defined as ≥1 inpatient diagnosis code | | |

#

## **Table II:** List of clinical measurements extracted, associated counts of gold-standard clinician annotations

| **Feature** | **Unit** | **Clinician Annotation Count** |
| --- | --- | --- |
| **Left ventricle anatomy and function** | | |
| Left ventricular end diastolic volume | cc | 311 |
| Left ventricular end diastolic volume index | cc/m2 | 317 |
| Left ventricular end diastolic diameter | mm | 196 |
| Left ventricular end systolic volume | cc | 317 |
| Left ventricular end systolic volume index | cc/m2 | 230 |
| Left ventricular end systolic diameter | mm | 222 |
| Left ventricular ejection fraction | % | 497 |
| Left ventricular stroke volume | cc | 349 |
| Left ventricular mass | g | 148 |
| Left ventricular mass index | g/m2 | 304 |
| Cardiac output | L/min | 174 |
| Cardiac index | L/min/m2 | 162 |
| **Right ventricle anatomy and function** | | |
| Right ventricular end diastolic volume | cc | 280 |
| Right ventricular end diastolic volume index | cc/m2 | 318 |
| Right ventricular end systolic volume | cc | 289 |
| Right ventricular end systolic volume index | cc/m2 | 210 |
| Right ventricular stroke volume | cc | 316 |
| Right ventricular ejection fraction | % | 405 |
| **Other cardiac structural anatomy** | | |
| Left atrial anterior-posterior dimension | mm | 257 |
| Aortic root dimension | mm | 254 |
| Pulmonary artery dimension | mm | 257 |

## **Table III:** Inter-Annotator Agreement Across Measurements in the Test Sample (n=100 Reports)

| **Feature** | **Inter-Annotator Agreement (%)** |
| --- | --- |
| **Left ventricle anatomy and function** | |
| Left ventricular end diastolic volume | 98.8% |
| Left ventricular end diastolic volume index | 95.9% |
| Left ventricular end diastolic diameter | 97.9% |
| Left ventricular end systolic volume | 100.0% |
| Left ventricular end systolic volume index | 97.9% |
| Left ventricular end systolic diameter | 97.9% |
| Left ventricular ejection fraction | 98.9% |
| Left ventricular stroke volume | 98.8% |
| Left ventricular mass | 84.6% |
| Left ventricular mass index | 89.4% |
| Cardiac output | 91.3% |
| Cardiac index | 90.5% |
| **Right ventricle anatomy and function** | |
| Right ventricular end diastolic volume | 98.6% |
| Right ventricular end diastolic volume index | 87.1% |
| Right ventricular end systolic volume | 97.2% |
| Right ventricular end systolic volume index | 91.1% |
| Right ventricular stroke volume | 94.3% |
| Right ventricular ejection fraction | 93.1% |
| **Other cardiac structural anatomy** | |
| Left atrial anterior-posterior dimension | 51.6% |
| Aortic root dimension | 76.7% |
| Pulmonary artery dimension | 76.5% |

## **Table IV:** Upper and Lower Bounds to Filter Model Extractions

| **Feature** | **Unit** | **Lower Bound** | **Upper Bound** |
| --- | --- | --- | --- |
| **Left ventricle anatomy and function** | | |  |
| Left ventricular end diastolic volume | cc | 10.0 | 583.0 |
| Left ventricular end diastolic volume index | cc/m2 | 10.0 | 309.0 |
| Left ventricular end diastolic diameter | mm | 10.0 | 93.0 |
| Left ventricular end systolic volume | cc | 10.0 | 502.0 |
| Left ventricular end systolic volume index | cc/m2 | 10.0 | 269.0 |
| Left ventricular end systolic diameter | mm | 10.0 | 93.0 |
| Left ventricular ejection fraction | % | 5.0 | 90.0 |
| Left ventricular stroke volume | cc | 10.0 | 228.0 |
| Left ventricular mass | g | 30.0 | 400.0 |
| Left ventricular mass index | g/m2 | 20.0 | 225.0 |
| Cardiac output | L/min | 2.0 | 20.0 |
| Cardiac index | L/min/m2 | 0.5 | 10.0 |
| **Right ventricle anatomy and function** | | |  |
| Right ventricular end diastolic volume | cc | 10.0 | 340.0 |
| Right ventricular end diastolic volume index | cc/m2 | 10.0 | 186.0 |
| Right ventricular end systolic volume | cc | 10.0 | 255.0 |
| Right ventricular end systolic volume index | cc/m2 | 10.0 | 134.0 |
| Right ventricular stroke volume | cc | 17.0 | 173.0 |
| Right ventricular ejection fraction | % | 5.0 | 90.0 |
| **Other cardiac structural anatomy** | | |  |
| Left atrial anterior-posterior dimension | mm | 10.0 | 90.0 |
| Aortic root dimension | mm | 20.0 | 50.0 |
| Pulmonary artery dimension | mm | 10.0 | 50.0 |

## **Table V:** Feature-level performance for BERT_LARGE_, fine-tuned for 12 epochs, evaluated on 100 reports annotated with gold-standard clinician labels.

| **Feature** | **F-1 Score** |
| --- | --- |
| Macro-Average, all features | 0.957 |
| **Left ventricle anatomy and function** | |
| Left ventricular end diastolic volume | 0.982 |
| Left ventricular end diastolic volume index | 0.951 |
| Left ventricular end diastolic diameter | 0.979 |
| Left ventricular end systolic volume | 0.976 |
| Left ventricular end systolic volume index | 1.000 |
| Left ventricular end systolic diameter | 1.000 |
| Left ventricular ejection fraction | 0.924 |
| Left ventricular stroke volume | 0.959 |
| Left ventricular mass | 0.950 |
| Left ventricular mass index | 0.969 |
| Cardiac output | 0.989 |
| Cardiac index | 0.988 |
| **Right ventricle anatomy and function** | |
| Right ventricular end diastolic volume | 0.993 |
| Right ventricular end diastolic volume index | 0.978 |
| Right ventricular end systolic volume | 0.993 |
| Right ventricular end systolic volume index | 0.957 |
| Right ventricular stroke volume | 0.958 |
| Right ventricular ejection fraction | 0.958 |
| **Other cardiac structural anatomy** | |
| Left atrial anterior-posterior dimension | 0.919 |
| Aortic root dimension | 0.902 |
| Pulmonary artery dimension | 0.962 |

Note: This represents performance prior to post-processing and filtering for physiological limits

## **Table VI:** Results of filtering model extractions by physiologic lower- and upper- bounds

| **Feature** | **Within Range** | **Below Range** | **Above Range** |
| --- | --- | --- | --- |
| **Left ventricle anatomy and function** | | |  |
| Left ventricular end diastolic volume | 99.50 | 0.28 | 0.22 |
| Left ventricular end diastolic volume index | 98.69 | 1.15 | 0.16 |
| Left ventricular end diastolic diameter | 99.16 | 0.36 | 0.27 |
| Left ventricular end systolic volume | 99.32 | 0.45 | 0.23 |
| Left ventricular end systolic volume index | 95.72 | 3.75 | 0.53 |
| Left ventricular end systolic diameter | 99.38 | 0.37 | 0.25 |
| Left ventricular ejection fraction | 99.49 | 0.14 | 0.37 |
| Left ventricular stroke volume | 99.51 | 0.21 | 0.28 |
| Left ventricular mass | 95.58 | 1.28 | 0.14 |
| Left ventricular mass index | 94.86 | 4.91 | 0.23 |
| Cardiac output | 98.78 | 0.76 | 0.47 |
| Cardiac index | 85.37 | 2.87 | 11.77 |
| **Right ventricle anatomy and function** | | |  |
| Right ventricular end diastolic volume | 98.95 | 0.10 | 0.95 |
| Right ventricular end diastolic volume index | 97.95 | 1.35 | 0.70 |
| Right ventricular end systolic volume | 98.81 | 0.41 | 0.78 |
| Right ventricular end systolic volume index | 98.08 | 1.51 | 0.41 |
| Right ventricular stroke volume | 99.25 | 0.25 | 0.50 |
| Right ventricular ejection fraction | 99.28 | 0.24 | 0.48 |
| **Other cardiac structural anatomy** | | |  |
| Left atrial anterior-posterior dimension | 98.73 | 0.82 | 0.49 |
| Aortic root dimension | 96.74 | 1.49 | 1.44 |
| Pulmonary artery dimension | 97.48 | 1.28 | 1.08 |

#

All values in % of extractions

## **Table VII:** Counts and distribution metrics for model extracted measurements across the study cohort

| **Feature** | **Unit** | **Count** | **Mean** | **SD** |
| --- | --- | --- | --- | --- |
| **Left ventricle anatomy and function** | | |  |  |
| Left ventricular end diastolic volume | cc | 9,042 | 171.48 | 66.90 |
| Left ventricular end diastolic volume index | cc/m2 | 6,716 | 87.84 | 32.82 |
| Left ventricular end diastolic diameter | mm | 5,564 | 55.32 | 9.41 |
| Left ventricular end systolic volume | cc | 9,108 | 84.24 | 60.21 |
| Left ventricular end systolic volume index | cc/m2 | 3,781 | 43.35 | 28.52 |
| Left ventricular end systolic diameter | mm | 5,133 | 40.50 | 10.83 |
| Left ventricular ejection fraction | % | 10,023 | 53.53 | 14.58 |
| Left ventricular stroke volume | cc | 8,893 | 87.11 | 28.06 |
| Left ventricular mass | g | 4,308 | 130.71 | 51.32 |
| Left ventricular mass index | g/m2 | 6,068 | 64.92 | 21.99 |
| Cardiac output | L/min | 5,094 | 6.11 | 1.94 |
| Cardiac index | L/min/m2 | 2,829 | 3.13 | 1.02 |
| **Right ventricle anatomy and function** | | |  |  |
| Right ventricular end diastolic volume | cc | 7,814 | 155.28 | 50.41 |
| Right ventricular end diastolic volume index | cc/m2 | 6,008 | 64.92 | 21.99 |
| Right ventricular end systolic volume | cc | 7,866 | 78.71 | 36.64 |
| Right ventricular end systolic volume index | cc/m2 | 3,313 | 38.59 | 16.29 |
| Right ventricular stroke volume | cc | 7,445 | 76.73 | 24.74 |
| Right ventricular ejection fraction | % | 8,532 | 50.69 | 11.59 |
| **Other cardiac structural anatomy** | | |  |  |
| Left atrial anterior-posterior dimension | mm | 7,050 | 41.09 | 9.50 |
| Aortic root dimension | mm | 6,236 | 31.46 | 5.79 |
| Pulmonary artery dimension | mm | 5,574 | 27.48 | 4.97 |

## **Table VIII:** Model-extracted measurement occurrence across reports in study cohort, in reference to gold standard annotations

| **Feature** | **Gold Standard Occurrence** | **Model Extractions** | | |
| --- | --- | --- | --- | --- |
|  |  | **Instances** | **Reports** | **Occurrence** |
| **Left ventricle anatomy and function** | | |  |  |
| Left ventricular end diastolic volume | 67.78 | 9,042 | 8,820 | 69.31 |
| Left ventricular end diastolic volume index | 59.78 | 6,716 | 6,505 | 51.12 |
| Left ventricular end diastolic diameter | 41.56 | 5,564 | 5,320 | 41.80 |
| Left ventricular end systolic volume | 67.56 | 9,108 | 8,721 | 68.53 |
| Left ventricular end systolic volume index | 40.00 | 3,781 | 3,704 | 29.11 |
| Left ventricular end systolic diameter | 40.22 | 5,133 | 5,051 | 39.69 |
| Left ventricular ejection fraction | 73.78 | 10,023 | 9,183 | 72.16 |
| Left ventricular stroke volume | 65.78 | 8,893 | 8,710 | 68.44 |
| Left ventricular mass | 30.44 | 4,308 | 4,164 | 32.72 |
| Left ventricular mass index | 51.33 | 6,068 | 5,951 | 46.76 |
| Cardiac output | 38.44 | 5,094 | 4,791 | 37.65 |
| Cardiac index | 36.00 | 2,829 | 2,803 | 22.03 |
| **Right ventricle anatomy and function** | | |  |  |
| Right ventricular end diastolic volume | 61.56 | 7,814 | 7,651 | 60.12 |
| Right ventricular end diastolic volume index | 54.44 | 6,008 | 5,846 | 45.94 |
| Right ventricular end systolic volume | 60.22 | 7,866 | 7,655 | 60.15 |
| Right ventricular end systolic volume index | 36.67 | 3,313 | 3,269 | 25.69 |
| Right ventricular stroke volume | 57.11 | 7,445 | 7,348 | 57.74 |
| Right ventricular ejection fraction | 57.11 | 8,532 | 7,753 | 60.92 |
| **Other cardiac structural anatomy** | | |  |  |
| Left atrial anterior-posterior dimension | 52.67 | 7,050 | 6,767 | 53.17 |
| Aortic root dimension | 45.33 | 6,236 | 5,856 | 46.02 |
| Pulmonary artery dimension | 44.67 | 5,574 | 5,187 | 40.76 |

**References**

1. [string — Common string operations — Python 3.10.5 documentation. [cited 28 Jun 2022]. Available:](http://paperpile.com/b/80qaDl/FfQEa) <https://docs.python.org/3/library/string.html>

2. [num2words: Modules to convert numbers to words. 42 --> forty-two. Github; Available:](http://paperpile.com/b/80qaDl/SDDhR) <https://github.com/savoirfairelinux/num2words>
